# Development and validation of an AMR-based predictive model for post-PCI upper gastrointestinal bleeding in NSTEMI patients

**DOI:** 10.3389/fendo.2025.1545462

**Published:** 2025-04-16

**Authors:** Zhaokai Wang, Shuping Yang, Chunxue Zhou, Cheng Li, Chengcheng Chen, Junhong Chen, Dongye Li, Lei Li, Tongda Xu

**Affiliations:** ^1^ Department of Cardiology, The Affiliated Hospital of Xuzhou Medical University, Xuzhou, Jiangsu, China; ^2^ Department of General Practice, The Affiliated Hospital of Xuzhou Medical University, Xuzhou, Jiangsu, China; ^3^ Institute of Cardiovascular Disease Research, Xuzhou Medical University, Xuzhou, Jiangsu, China

**Keywords:** angiographic microvascular resistance of culprit vessel, upper gastrointestinal bleeding, non-ST-segment elevation myocardial infarction, nomogram, percutaneous coronary intervention

## Abstract

**Background:**

Upper gastrointestinal bleeding (UGIB) is a common complication in patients with non-ST-segment elevation myocardial infarction (NSTEMI) after percutaneous coronary intervention (PCI), and the aim of our study is to construct a nomogram for predicting the occurrence of UGIB within 1 year after PCI in NSTEMI patients.

**Methods:**

In this study, 784 patients with NSTEMI after PCI in the Affiliated Hospital of Xuzhou Medical University between September 1, 2017 and August 31, 2019 were included as the training group, and 336 patients from the East Affiliated Hospital of Xuzhou Medical University were included as the external validation group. Classical regression methods were combined with a machine learning model to identify the independent risk factors. These factors based on multivariate logistic regression analysis were then utilized to develop a nomogram. The performance of the nomogram was evaluated using the area under the receiver operating characteristic curve (AUC), calibration plots, and decision curve analysis (DCA).

**Results:**

The nomogram consisted of six independent predictors, including HASBLED, triglyceride glucose index, alcohol drinking, red blood cell count, use of proton pump inhibitor, and angiographic microvascular resistance of culprit vessel. Training and validation groups accurately predicted the occurrence of UGIB (AUC, 0.936 and 0.910). The calibration curves showed that the nomogram agreed with the actual observations and the DCA also demonstrated that the nomogram was applicable in the clinic.

**Conclusion:**

We developed a simple and effective nomogram for predicting the occurrence of UGIB within 1 year in NSTEMI patients after PCI based on angiographic microvascular resistance.

## Introduction

Coronary artery disease (CAD) is the leading major cause of death and loss of healthy life in society, both in terms of morbidity, mortality, and disease progression (1). Non-ST-segment elevation myocardial infarction (NSTEMI) is a severe type of CAD and in NSTEMI, coronary atherosclerotic plaque erosion, rupture or subsequent thrombosis causes partial obstruction of the coronary arteries, which can lead to myocardial necrosis (2). In myocardial infarction, NSTEMI accounted for 63.1% and 4.2% of NSTEMI patients died in the hospital (3).

With advances in antiplatelet and percutaneous coronary intervention (PCI) therapies, cardiovascular morbidity and mortality in patients with NSTEMI have decreased significantly (4). However, the occurrence of gastrointestinal bleeding (GIB) after PCI remains a major problem. The most common bleeding after PCI was GIB, which accounted for 61.7% of all bleeding (5). A study indicated that the incidence of upper gastrointestinal bleeding (UGIB) in patients with ACS was 8.9% within 30 days of PCI, 4.7% within 1 year, and rose to 10.1% beyond 1 year (6). Yasuda et al. found that the incidence of UGIB at 1 and 2 years after PCI was 2.5% and 5%, respectively, whereas the incidence was higher in patients who did not use the proton pump inhibitor (PPI), at 4.5% and 9.2%, respectively (7). And patients with acute coronary syndrome (ACS) had a 62% mortality rate for UGIB compared to patients with UGIB only (8). This caused a certain burden on both the cost of hospitalization and medical insurance. Thus, early screening for UGIB in ACS patients is necessary.

Coronary microcirculatory disorders (CMD) is disease that affect the structure and function of the coronary microcirculation. There were various methods of assessing CMD, categorized as invasive and non-invasive. Positron emission computed tomography (PET) was considered the gold standard for noninvasive assessment of CMD by quantifying myocardial blood flow (MBF) and assessing myocardial perfusion reserve (9). Cardiac magnetic resonance imaging (CMR) and myocardial contrast echocardiography could also noninvasively quantify MBF (10). Invasive assessment methods such as coronary angiography could assess microvascular dilatation by coronary flow reserve (CFR) (10). The index of microcirculatory resistance (IMR) was the gold standard for measuring CMD and was obtained by measuring the product of distal coronary artery pressure and the mean passage time of saline push during maximal congestion induced by adenosine or opioid (11). In recent years, angiographic microvascular resistance (AMR) has been proposed for the assessment of CMD and was mainly calculated by quantitative flow ratio (QFR) (12). Compared with invasive methods, AMR did not rely on drug induction such as adenosine and require additional operations in clinical applications, providing simplicity and maneuverability. And it enabled accurate numerical assessment of microvascular function at a lower cost than non-invasive methods. Hence, it was considered an emerging method to CMD assessment.

Some chronic diseases (e.g., heart failure, hypertension, diabetes, metabolic syndrome, cardiac hypertrophy, and CAD) and post-PCI were risk factors for CMD (13–16). We pondered whether CMD affected systemic microcirculation and would increase the risk of having UGIB. Therefore, we constructed an AMR-based nomogram to predict UGIB within 1 year in NSTEMI patients after PCI.

## Materials and methods

### Study population and design

**Figure 1:**
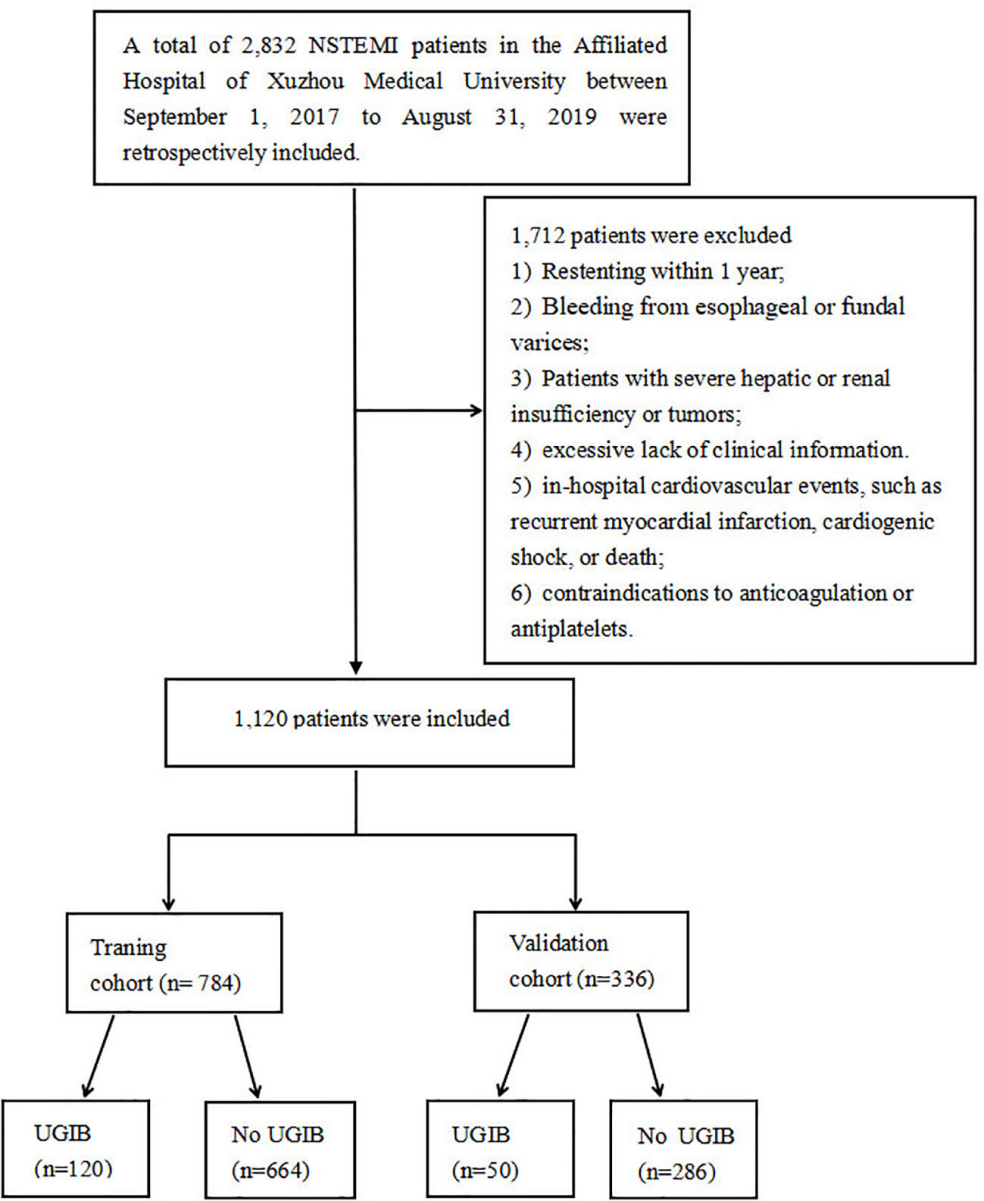
The study flowchart for developing and validating nomogram.

This study was approved by the Medical Research Ethics Committee of the Affiliated Hospital of Xuzhou Medical University (XYFY2023-KL043-01), which was conducted following the Declaration of Helsinki. Because our study was retrospective, the committee waived the requirement for written informed consent. Based on inclusion and exclusion criteria, we included patients with NSTEMI who underwent PCI from September 1, 2017, to August 31, 2019 in the Affiliated Hospital of Xuzhou Medical University (training group, n=784), and the East Hospital of Xuzhou Medical University (validation group, n=336). The flow chart of the study is shown in **Figure 1**.

Inclusion criteria: (1) Diagnosis of NSTEMI; (2) Successful implantation of the drug-eluting stent; (3) Readmission for UGIB within 1 year after PCI; and (4) over 18 years old.

Meanwhile, exclusion criteria: (1) Restenting within 1 year; (2) Bleeding from esophageal or fundal varices ; (3) Patients with severe hepatic or renal insufficiency or tumors; (4) excessive lack of clinical information; (5) in-hospital cardiovascular events, such as recurrent myocardial infarction, cardiogenic shock, or death; (6) contraindications to anticoagulation or antiplatelets.

### Clinical treatment process

All patients with NSTEMI in this study underwent PCI in the digital subtraction angiography (DSA) suite, and patients were anticoagulated with heparin subcutaneously and given 300 mg of aspirin, 300 mg of clopidogrel, or 180 mg of ticagrelor orally as a loading dose before PCI. The various devices, instruments, and adjunctive medications (nitroglycerin, sodium nitroprusside, tirofiban, atropine, etc.) used during the procedure were determined by the operator based on the patient’s intraoperative condition.

Postoperatively, medications were prescribed according to guidelines. These therapies included (1) dual antiplatelet therapy (DAPT), including aspirin (100 mg once daily) in combination with ticagrelor (90 mg twice daily) or clopidogrel (75 mg once daily) (2); statin (3); beta-blocker; and (4) angiotensin-converting enzyme inhibitor/angiotensin receptor antagonist (ACEI/ARB).

### Collection of variables

Demographic data included age, sex, smoking, and drinking status. Previous history contained heart failure (HF), diabetes, stroke, hypertension, chronic kidney disease (CKD), bleeding, peripheral vascular disease (PVD), atrial fibrillation (AF), and peptic ulcer. In the history of bleeding, 16 patients had nosebleeds and 12 patients had cerebral hemorrhage before PCI. Physical examination contained body mass index (BMI), diastolic blood pressure (DBP), heart rate (HR), and systolic blood pressure (SBP). Laboratory tests contained white blood cell (WBC) count, hemoglobin (Hb), lymphocyte count, red blood cell (RBC) count, platelet (PLT) count, neutrophil, serum uric acid (sUA), C-reactive protein (CRP), estimated glomerular filtration rate (eGFR), glucose, glycated hemoglobin (HbA1c), serum creatinine (sCr), low-density lipoprotein (LDL), total cholesterol (TC), high-density lipoprotein (HDL), lipoprotein a (LPa), triglycerides (TG), N-terminal pro-brain natriuretic peptide (NT-proBNP), international normalized ratio (INR), left ventricular ejection fraction (LVEF), creatinekinase-MB (CKMB), fibrinogen (FIB), and total bilirubin(TBIL). Image data during PCI contained the culprit vessels (right coronary artery (RCA), left anterior descending branch (LAD), left circumflex branch (LCX)), multivessel disease, the number of stents, stent length, stent diameter, and Killip grade. Postoperative medications comprised of angiotensin-converting enzyme inhibitor/angiotensin receptor antagonist (ACEI/ARB), calcium antagonist (CCB), Low molecular weight heparin (LMWH), diuretics, proton pump inhibitor (PPI), beta-blockers, and nonsteroidal anti-inflammatory drug (NSAID).

Triglyceride glucose index (TyG) was calculated as Ln[TG(mg/dL) × FBG(mg/dL)/2]. HAS-BLED scores up to 9 points including hypertension, stroke, history of bleeding, abnormal liver/kidney function, labile INR, elderly, and drug/alcohol.

### Computation of AMR

AMR analysis was performed independently by certified technicians using commercial software (AngioPlus Core, Pulse Medical Imaging Technology Co., Ltd., Shanghai, China), who were blinded to the clinical data. Coronary artery image analysis was performed using the above system. The blood flow velocity was derived by dividing the vessel centerline length by the contrast fill time. Using high blood flow as a boundary condition, the pressure drop was calculated from the hydrodynamic equations. Distal coronary pressure (Pd) was calculated from the pressure drop, and μQFR was calculated by dividing Pd by mean aortic pressure (Pa). Angiography microvascular resistance (AMR) is computed as Pd divided by the hyperaemic flow velocity Velocity_hyp_ (17).


$$\text{AMR}=\frac{P_{d}}{{\text{Velocity}}_{hyp}}=\frac{P_{a}*\text{μQFR}}{{\text{Velocity}}_{hyp}}$$

### Clinical endpoints and definitions

The clinical endpoint was NSTEMI patients who were readmitted with UGIB symptoms within 1 year after the PCI. The definition of UGIB was clinical signs of coffee-ground vomiting, hematemesis, melena, or endoscopic findings of active bleeding from upper gastrointestinal sites.

### Statistical analysis

Categorical variables were expressed as frequencies and percentages and compared using the chi-square test or Fisher’s exact test. Continuous variables are expressed as mean ± standard deviation. Normally distributed variables were compared using the t-test for comparison, while non-normally distributed variables were compared using the Mann-Whitney U-test. The percentage of missing values were less than 20%, and multiple imputation was used to impute the missing data for the covariates. We first used univariate analysis and random forest to predict independent risk factors. The random forest algorithm generated the mean decreased Gini (MDG) that was used to reflect the contribution of each independent variable to the dependent variable. In the random forest algorithm, Ntree specifies the number of decision trees in the random forest and Brenneman suggests that the optimal number of decision trees is 500. Optimal mtry parameter was selected by grid search method, and then combined with different ntree to find the lowest out-of-bag error (OOB) rate. Finally, we included independent risk factors that were statistically significant in the univariate analysis (P< 0.01) and the top ten variables of the random forest MDG in the analysis. The variables were screened and used to perform the multivariate logistic regression model. The least absolute shrinkage and selection operator (LASSO) regression was then performed to ensure that the model was not overfitted. Finally, a nomogram of the multivariate model based on optimal predictors was developed to predict the probability of UGIB within 1 year after PCI. The consistency index (C-index) is the area under the curve (AUC) of the receiver operating characteristic curve (ROC) in logistic regression analysis for the discrimination capacity of the nomogram. The nomogram’s predictive accuracy was evaluated using calibration plots and the consistency between predicted and actual probabilities was assessed by the Hosmer-Lemeshow test. Clinical efficacy was evaluated using decision curve analysis (DCA). Data were analyzed using R Studio (Version 4.2.3, https://www.Rproject.org). P values < 0.05 were significant for all statistical tests.

### Ethics approval and consent to participate

This study was approved by the Medical Research Ethics Committee of the Affiliated Hospital of Xuzhou Medical University (XYFY2023-KL043-01), which was conducted following the Declaration of Helsinki. Because our study was retrospective, the Medical Research Ethics Committee of the Affiliated Hospital of Xuzhou Medical University waived the requirement for written informed consent.

## Results

### Participants characteristics

Our study included 1,120 NSTEMI patients after PCI from September 1, 2017, to August 31, 2019, from the Affiliated Hospital of Xuzhou Medical University and the East Affiliated Hospital of Xuzhou Medical University. Baseline characteristics were shown in **Table 1**. The average age of this study was 68.88 years old and most of the patients (68.8%) were male. There were 120 and 50 UGIB participants in the training and validation groups, respectively.

**Table 1:** Patient characteristics.

Variables	Validation Cohort (N=336)	Training Cohort (N=784)	P value
UGIB, n (%)			
No	286 (85.1)	664 (84.7)	0.856
Yes	50 (14.9)	120 (15.3)	
Age, years	68.56 (10.81)	69.02 (10.91)	0.516
Sex (%)			
Male, n (%)	237 (70.5)	533 (68.0)	0.399
Female, n (%)	99 (29.5)	251 (32.0)	
BMI, kg/m^2^	25.12 (3.62)	25.41 (3.68)	0.234
SBP, mmHg	129.94 (22.35)	133.29 (22.09)	0.021
DBP, mmHg	77.93 (12.92)	79.00 (13.52)	0.216
HR, times/min	75.68 (12.99)	74.95 (12.10)	0.362
Smoking, n (%)			
No	225 (67.0)	528 (67.3)	0.901
Yes	111 (33.0)	256 (32.7)	
Drinking, n (%)			
No	241 (71.7)	571 (72.8)	0.704
Yes	95 (28.3)	213 (27.2)	
Hypertension, n (%)			
No	167 (49.7)	370 (47.2)	0.441
Yes	169 (50.3)	414 (52.8)	
Diabetes, n (%)			
No	250 (74.4)	557 (71.0)	0.251
Yes	86 (25.6)	227 (29.0)	
CKD, n (%)			
No	319 (94.9)	747 (95.3)	0.808
Yes	17 (5.1)	37 (4.7)	
AF, n (%)			
No	319 (94.9)	757 (96.6)	0.202
Yes	17 (5.1)	27 (3.4)	
HF, n (%)			
No	297 (88.4)	658 (83.9)	0.053
Yes	39 (11.6)	126 (16.1)	
Stroke, n (%)			
No	265 (78.9)	636 (81.1)	0.384
Yes	71 (21.1)	148 (18.9)	
Bleeding, n (%)			
No	330 (98.2)	762 (97.2)	0.316
Yes	6 (1.8)	22 (2.8)	
HASBLED	2.67 (1.14)	2.65 (1.14)	0.814
Peptic Ulcer, n (%)			
No	295 (87.8)	714 (91.1)	0.093
Yes	41 (12.2)	70 (8.9)	
PVD, n (%)			
No	330 (98.2)	773 (98.6)	0.631
Yes	6 (1.8)	11 (1.4)	
Laboratory test			
WBC, ×10^9^/L	8.81 (2.85)	9.03 (3.55)	0.315
N, ×10^9^/L	6.67 (2.86)	7.04 (5.18)	0.221
L, ×10^9^/L	1.51 (0.81)	1.54 (0.83)	0.654
RBC, ×10^9^/L	4.40 (0.65)	4.33 (0.72)	0.182
Hb, g/L	136.13 (17.09)	135.19 (18.15)	0.421
PLT, ×10^9^/L	206.59 (62.46)	205.99 (65.70)	0.886
CRP, mg/dl	10.50 (26.78)	12.13 (31.10)	0.402
sCr, mmol/L	75.18 (77.57)	74.70 (75.88)	0.923
sUA, umol/L	309.89 (95.49)	311.26 (124.31)	0.857
eGFR, mL/min/ 1,73m^2^	107.49 (23.58)	108.13 (24.01)	0.679
Glucose, mmol/L	6.71 (3.00)	6.68 (2.74)	0.877
HbA1c (%)	6.56 (1.57)	6.57 (1.49)	0.968
TyG	7.26 (0.64)	7.27 (0.65)	0.698
TC, mmol/L	2.99 (1.74)	3.09 (1.80)	0.380
TG, mmol/L	1.48 (1.20)	1.54 (1.21)	0.473
HDL, mmol/L	2.19 (1.27)	2.19 (1.18)	0.991
LDL, mmol/L	119.72 (224.42)	117.40 (200.45)	0.803
LPa, mg/L	131.28 (199.53)	148.17 (247.39)	0.269
INR	0.97 (0.11)	0.99 (0.12)	0.053
LVEF, (%)	55.06 (8.47)	55.37 (8.37)	0.567
CKMB, ng/mL	50.06 (79.99)	48.73 (79.91)	0.799
NT-proBNP, pg/mL	1656.43 (3361.16)	2086.81 (4646.60)	0.125
FIB, g/L	3.25 (1.46)	3.66 (10.43)	0.470
TBIL, umol/L	15.10 (9.61)	15.10 (8.73)	0.999
Angiographic features			
Killip grade			
Grade I, n (%)	320 (95.2)	748 (95.4)	0.480
Grade II, n (%)	13 (3.9)	22 (2.8)	
Grade III, n (%)	1 (0.3)	8 (1.0)	
Grade IV, n (%)	2 (0.6)	6 (0.8)	
LAD, n (%)	158 (47.0)	351 (44.8)	0.488
LCX, n (%)	139 (41.4)	340 (43.4)	0.536
RCA, n (%)	114 (33.9)	274 (34.9)	0.742
Multivascular disease, n (%)			
No	291 (86.6)	689 (87.9)	0.554
Yes	45 (13.4)	95 (12.1)	
Stent diameter, mm	2.87 (0.45)	2.88 (0.47)	0.826
Stent length, mm	26.15 (7.23)	26.12 (7.12)	0.943
Number of stents, n (%)	1.49 (0.82)	1.39 (0.72)	0.040
Culprit Vessel AMR	2.23 (0.86)	2.23 (0.87)	0.988
Medication			
ACEI/ARB, n (%)			
No	176 (52.4)	370 (47.2)	0.111
Yes	160 (47.6)	414 (52.8)	
Beta-blocker, n (%)			
No	73 (21.7)	185 (23.6)	0.496
Yes	263 (78.3)	599 (76.4)	
LMWH, n (%)			
No	109 (32.4)	286 (36.5)	0.195
Yes	227 (67.6)	498 (63.5)	
CCB, n (%)			
No	290 (86.3)	648 (82.7)	0.128
Yes	46 (13.7)	136 (17.3)	
Diuretics, n (%)			
No	187 (55.7)	464 (59.2)	0.273
Yes	149 (44.3)	320 (40.8)	
PPI, n (%)			
No	51 (15.2)	116 (14.8)	0.869
Yes	285 (84.8)	668 (85.2)	
NASID, n (%)			
No	320 (95.2)	754 (96.2)	0.470
Yes	16 (4.8)	30 (3.8)	

UGIB, upper gastrointestinal bleeding; BMI, body mass index; SBP, systolic blood pressure; DBP, diastolic blood pressure; HR, heart rate; CKD, chronic kidney disease; AF, atrial fibrillation; HF, heart failure; HASBLED, including hypertension, abnormal liver/kidney function, stroke, history of bleeding, labile INR, elderly, and drug/alcohol; PVD, Peripheral vascular disease; WBC, white blood cell; N, neutrophils; L, lymphocytes; RBC, red blood cell; Hb, hemoglobin; PLT, platelets; CRP, C-reactive protein; sCr, serum creatinine; sUA, serum uric acid; eGFR, estimated glomerular filtration rate; HbA1c, glycated hemoglobin; TyG, triglyceride glucose index; TC, total cholesterol; TG, triglycerides; HDL, high-density lipoprotein; LDL, low density lipoprotein; INR, international normalized ratio; LVEF, left ventricular ejection fraction; CKMB, creatine kinase isoenzyme-MB; NT-proBNP, N-terminal pro-brain natriuretic peptide; FIB, fibrinogen; TBIL, total bilirubin; LAD, left anterior descending; LCX, left circumflex branch; RCA, right coronary artery; AMR, angiography-derived microcirculatory resistance; ACEI/ARB, angiotensin-converting enzyme inhibitors/angiotensin II receptor blockers; LMWH, low molecular weight heparin; CCB, calcium channel antagonist; PPI, proton pump inhibitor; NASID, nonsteroidal anti-inflammatory drug.

### Potential predictors of UGIB and construction of the nomogram

The results of the univariate analyses and random forest were displayed in **Table 2**. The default value of Ntree was 500. After testing and adjusting, mtry=6 and ntree=300 had the lowest rate of OOB (2.81%). These variables (HASBLED, TyG index, Alcohol drinking, RBC count, PPI use, and AMR of culprit vessel) not only had a significant difference in the univariate analysis (p< 0.01) but also obtained high MDG (top ten) in the random forest. Therefore, these six variables were included in further multivariate logistic regression model.

**Table 2:** Univariate logistic regression analysis in the training group.

Variables	OR (95%CI)	P value	MDG
HASBLED	3.410 (2.720,4.274)	<0.001	14.055
RBC, ×10^9^/L	0.269 (0.197,0.367)	<0.001	11.639
TyG	3.631 (2.617,5.038)	<0.001	8.655
Culprit Vessel AMR	2.374 (1.843,3.059)	<0.001	8.568
CRP, mg/dl	0.980 (0.964,0.996)	0.017	7.429
CKMB, ng/mL	0.998 (0.995,1.001)	0.201	7.398
Drinking, n (%)	7.621 (4.996,11.624)	<0.001	6.682
PPI, n (%)	0.139 (0.090,0.217)	<0.001	5.944
WBC, ×10^9^/L	0.921 (0.860,0.985)	0.017	5.531
FIB, g/L	1.003 (0.988,1.018)	0.688	5.042
LAD, n (%)	6.416 (3.994,10.305)	<0.001	4.666
NT-proBNP, pg/mL	1.000 (1.000,1.000)	0.001	4.663
N, ×10^9^/L	0.848 (0.785,0.918)	<0.001	4.492
LVEF, (%)	1.044 (1.018,1.071)	0.001	4.422
LDL, mmol/L	0.995 (0.992,0.997)	<0.001	4.413
HbA1c (%)	1.148 (1.023,1.288)	0.019	4.342
sCr, mmol/L	1.004 (1.001,1.006)	0.007	4.136
Hb, g/L	0.975 (0.965,0.986)	<0.001	3.728
eGFR, mL/min/1,73m^2^	0.984 (0.976,0.992)	<0.001	3.605
LPa, mg/L	1.001 (1.000,1.001)	0.085	3.492
TG, mmol/L	1.063 (0.925,1.221)	0.389	3.384
sUA, umol/L	1.001 (1.000,1.002)	0.120	3.355
TBIL, umol/L	0.968 (0.941,0.996)	0.027	3.316
BMI, kg/m^2^	0.922 (0.871,0.977)	0.006	3.184
TC, mmol/L	1.161 (1.043,1.293)	0.006	3.072
PLT, ×10^9^/L	1.001 (0.998,1.004)	0.511	3.046
HR, times/min	0.991 (0.975,1.008)	0.285	2.983
INR	2.991 (0.627,14.275)	0.169	2.864
Age, years	1.014 (0.995,1.033)	0.153	2.746
LMWH, n (%)	0.279 (0.187,0.419)	<0.001	2.740
HDL, mmol/L	0.753 (0.621,0.902)	0.003	2.511
Glucose, mmol/L	1.006 (0.938,1.079)	0.870	2.398
L, ×10^9^/L	0.724 (0.549,0.956)	0.023	2.320
Number of stents, n (%)	1.846 (1.467,2.322)	<0.001	2.294
Stent length, mm	1.010 (0.982,1.038)	0.485	2.203
NASID, n (%)	8.265 (3.915,17.847)	<0.001	2.071
SBP, mmHg	1.007 (0.998,1.016)	0.121	2.004
DBP, mmHg	1.003 (0.988,1.017)	0.727	1.943
Stent diameter, mm	0.761 (0.494,1.171)	0.214	1.877
CKD, n (%)	6.751 (3.427,13.299)	<0.001	1.273
Multivascular disease, n (%)	3.469 (2.145,5.610)	<0.001	1.167
AF, n (%)	6.614 (3.025,14.462)	<0.001	1.036
Smoking, n (%)	0.439 (0.271,0.710)	0.001	0.666
RCA, n (%)	0.960 (0.637,1.446)	0.845	0.647
Stroke, n (%)	2.008 (1.290,3.126)	0.002	0.607
Beta-blocker, n (%)	0.466 (0.307,0.705)	<0.001	0.597
ACEI/ARB, n (%)	0.613 (0.414,0.908)	0.015	0.507
Male, n (%)	1.171 (0.771,1.811)	0.468	0.485
HF, n (%)	1.758 (1.094,2.824)	0.020	0.484
Diuretics, n (%)	0.656 (0.435,0.991)	0.045	0.465
Diabetes, n (%)	1.112 (0.729,1.697)	0.622	0.446
CCB, n (%)	1.393 (0.862,2.251)	0.176	0.446
Bleeding, n (%)	3.316 (1.360,8.088)	0.008	0.414
LCX, n (%)	0.634 (0.422,0.952)	0.028	0.403
Hypertension, n (%)	1.813 (1.209,2.718)	0.004	0.361
Peptic Ulcer, n (%)	1.161 (0.603,2.234)	0.655	0.279
PVD, n (%)	6.937 (2.082,23.109)	0.002	0.257
Killip grade			0.202
Grade I, n (%)	Reference		
Grade II, n (%)	1.688 (0.610,4.668)	0.313	
Grade III, n (%)	3.443 (0.811,14.613)	0.094	
Grade IV, n (%)	1.148 (0.133,9.918)	0.900	

OR, Odds Ratio; MDG, mean decreased Gini; UGIB, upper gastrointestinal bleeding; BMI, body mass index; SBP, systolic blood pressure; DBP, diastolic blood pressure; HR, heart rate; CKD, chronic kidney disease; AF, atrial fibrillation; HF, heart failure; HASBLED, including hypertension, abnormal liver/kidney function, stroke, history of bleeding, labile INR, elderly, and drug/alcohol; PVD, Peripheral vascular disease; WBC, white blood cell; N, neutrophils; L, lymphocytes; RBC, red blood cell; Hb, hemoglobin; PLT, platelets; CRP, C-reactive protein; sCr, serum creatinine; sUA, serum uric acid; eGFR, estimated glomerular filtration rate; HbA1c, glycated hemoglobin; TyG, triglyceride glucose index; TC, total cholesterol; TG, triglycerides; HDL, high-density lipoprotein; LDL, low density lipoprotein; INR, international normalized ratio; LVEF, left ventricular ejection fraction; CKMB, creatine kinase isoenzyme-MB; NT-proBNP, N-terminal pro-brain natriuretic peptide; FIB, fibrinogen; TBIL, total bilirubin; LAD, left anterior descending; LCX, left circumflex branch; RCA, right coronary artery; AMR, angiography-derived microcirculatory resistance; ACEI/ARB, angiotensin-converting enzyme inhibitors/angiotensin II receptor blockers; LMWH, low molecular weight heparin; CCB, calcium channel antagonist; PPI, proton pump inhibitor; NASID, nonsteroidal anti-inflammatory drug.

Six potential risk factors were included in the multivariate logistic regression model (**Table 3**). In the training set model, a high HASBLED, TyG index, and AMR of the culprit vessel were correlated with developing the risk of UGIB (OR: 2.615, 95% CI: 1.940-3.620, P< 0.001; OR: 4.482, 95% CI: 2.813-7.366, P< 0.001; OR: 3.251, 95% CI: 2.216-4.899, P< 0. 001). Alcohol drinking was positively associated with the occurrence of UGIB (OR: 2.985, 95% CI: 1.584-5.665, P< 0.001). While taking PPI and higher RBC count played a protective role (OR: 0.179, 95% CI: 0.090-0.350, P< 0. 001; OR: 0.293, 95% CI: 0.188-0.439, P< 0. 001).

**Table 3:** Multivariate logistic regression analysis in training group.

Variables	OR	95%CI	P value
HASBLED	2.615	(1.940;3.620)	<0.001
TyG	4.482	(2.813;7.366)	<0.001
Drinking	2.985	(1.584;5.665)	<0.001
RBC	0.293	(0.188;0.439)	<0.001
PPI	0.179	(0.090;0.350)	<0.001
Culprit vessel AMR	3.251	(2.216;4.899)	<0.001

TyG, triglyceride glucose index; PPI, proton pump inhibitor; AMR, angiography-derived microcirculatory resistance.

**Figure 2:**
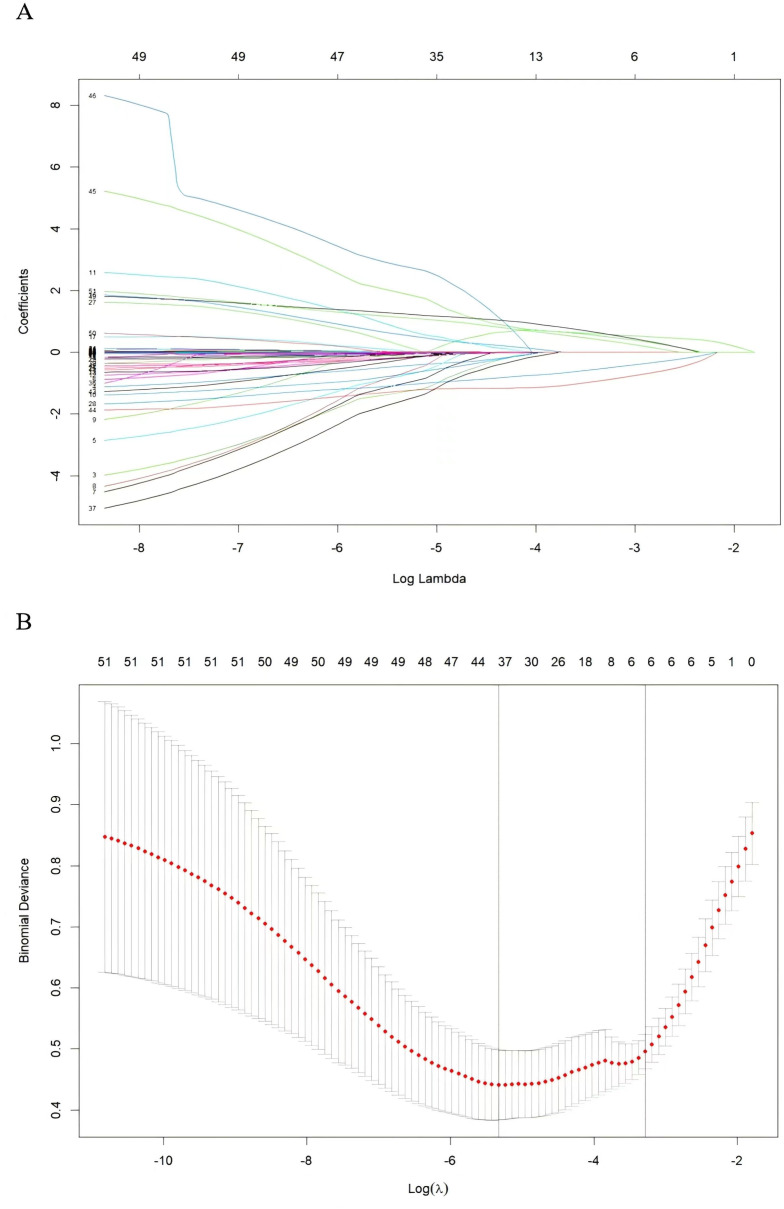
Variable screening based on lasso regression. **(A)** Characterization of the variation of variable coefficients; **(B)** The process of selecting the optimal value of the parameter λ in the Lasso regression model by the cross-validation method.

**Figure 3:**
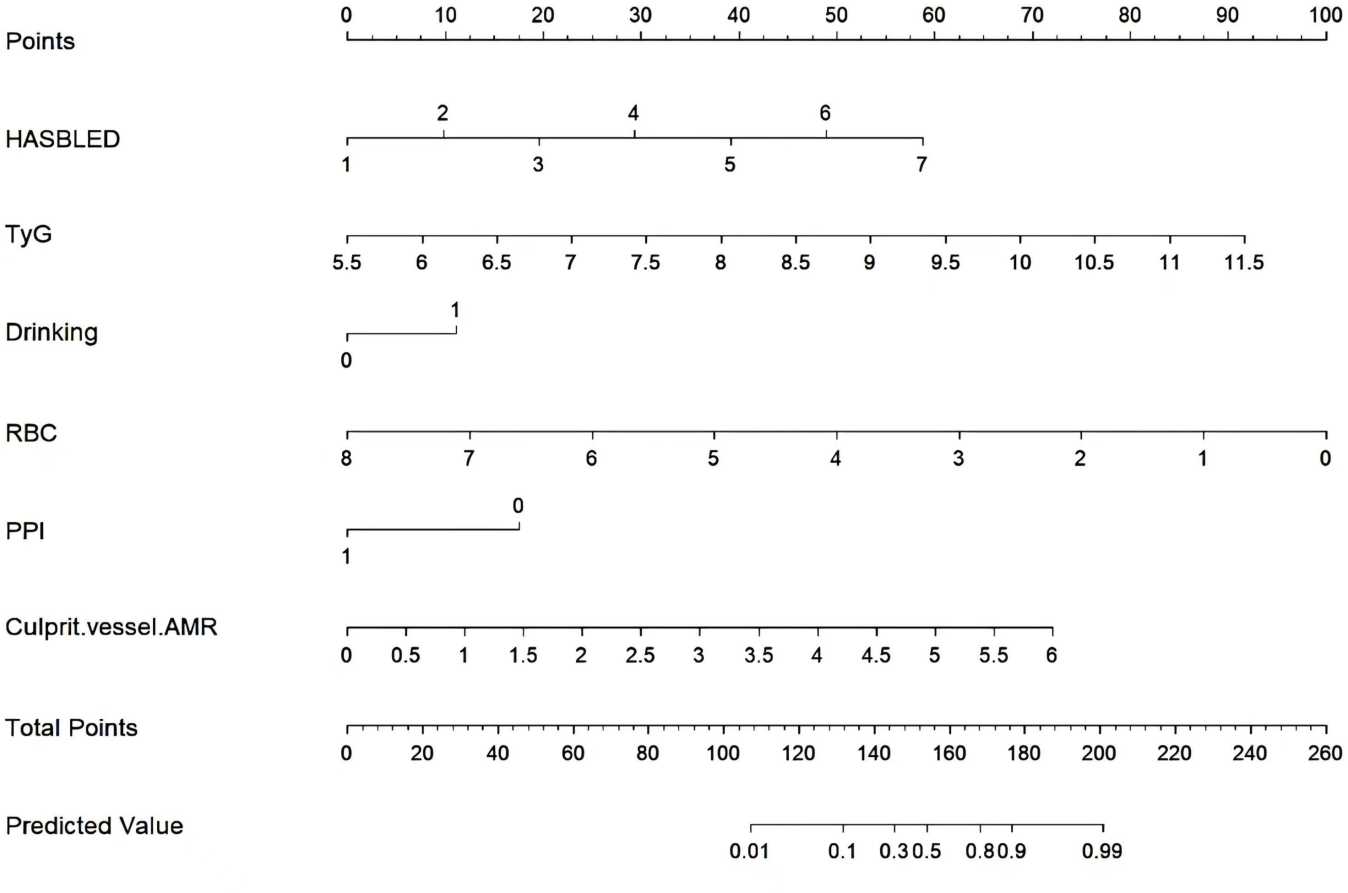
Nomogram for predicting the possibility of UGIB in NSTEMI patient after PCI.

Subsequently, these predictors were determined using the least absolute shrinkage and selection operator (LASSO) method for screening non-zero coefficient characteristics (**Figures 2A, B**). We used tenfold cross-validation to select the appropriate tuning parameters (λ) for the LASSO model. The optimal reconciliation coefficients λ.min at the minimum MSE and λ.1se at one standard MSE error were 0.004 and 0.031, respectively. The advantages of the Lasso regression were highly predictive and robust and minimized the effects of multicollinearity. Finally, the nomogram model was constructed using these six independent factors to predict the occurrence of UGIB within 1 year after PCI (**Figure 3**).

### Validation of the nomogram

**Figure 4:**
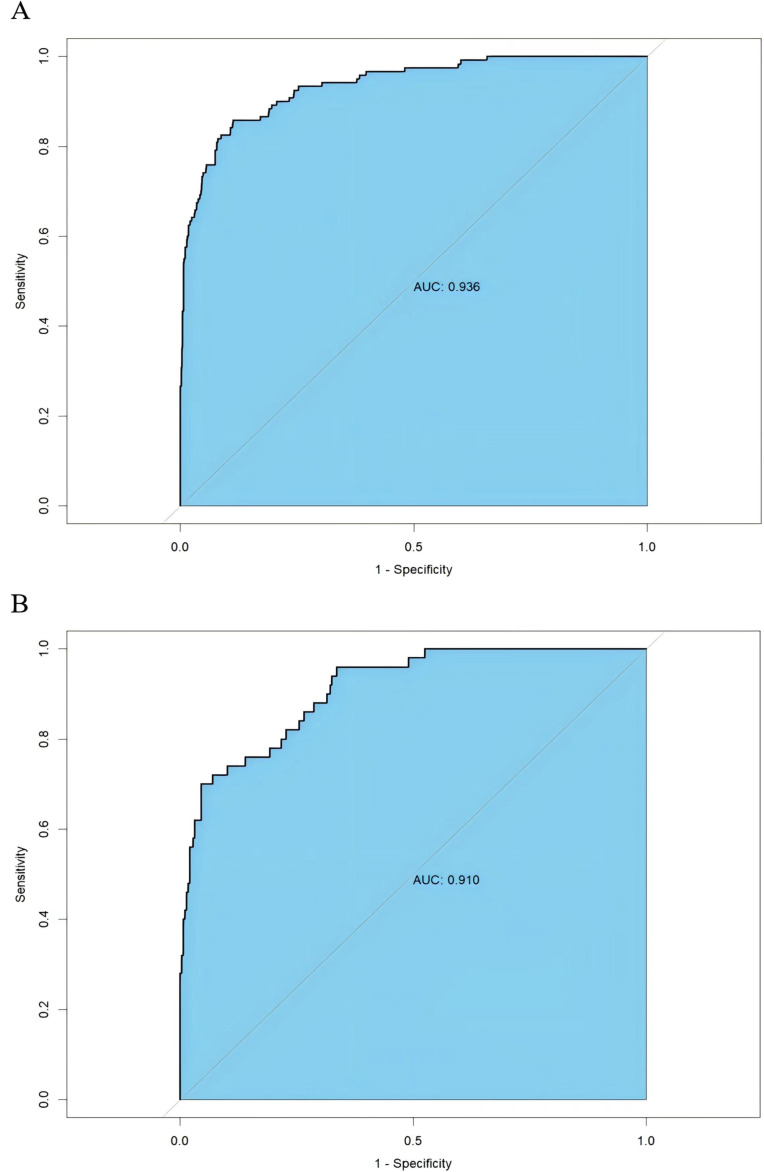
Receiver operating characteristics curve of the nomogram in the training group **(A)** and the validation group **(B)**.

**Figure 5:**
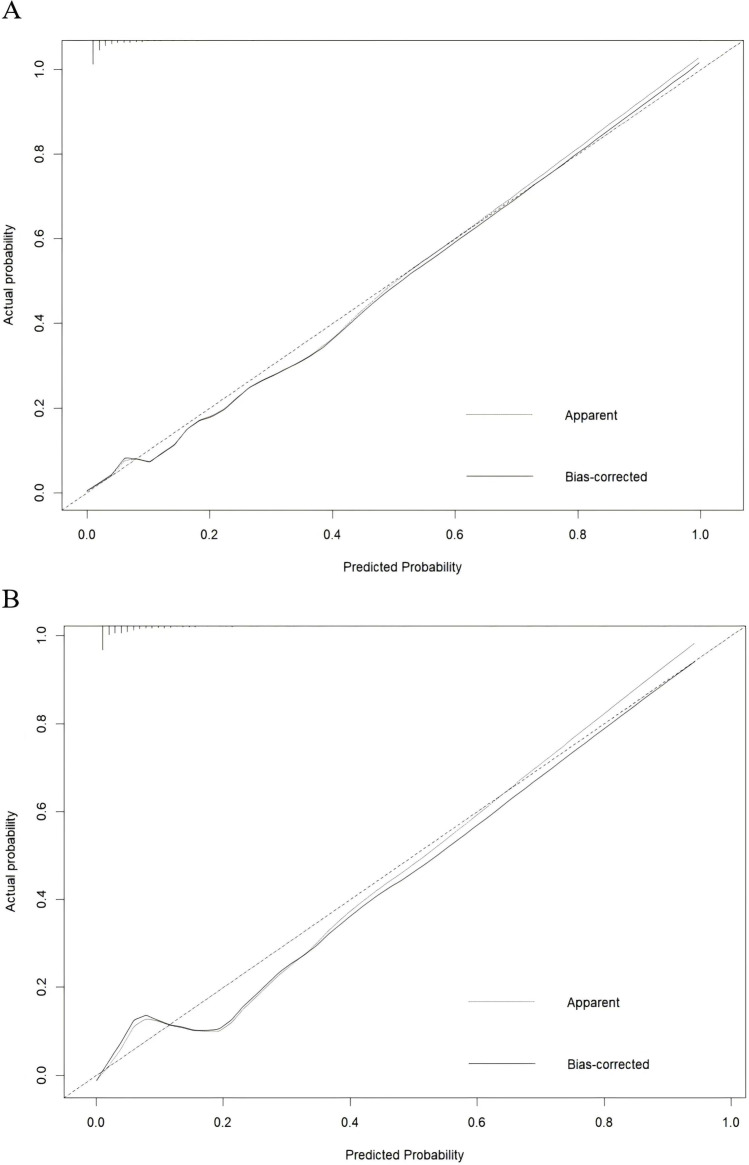
Calibration curve for the training group **(A)** and the validation group **(B)**, the horizontal axis denotes the overall predicted probability of UGIB in NSTEMI patients after percutaneous coronary intervention, and the vertical axis displays the actual probability.

The AUC of training and external validation groups was 0.936 and 0.910 in the ROC curve (**Figures 4A, B**), which demonstrated an outstanding discrimination of the nomogram. Calibration plots identified good consistency between the nomogram (**Figure 5A**) and the validation cohort (**Figure 5B**). The Hosmer-Lemeshow test indicated that the probability of UGIB after PCI predicted by the nomogram was consistent between the training set (P = 0.728) and the validation set (P = 0.269).

### Clinical use

**Figure 6:**
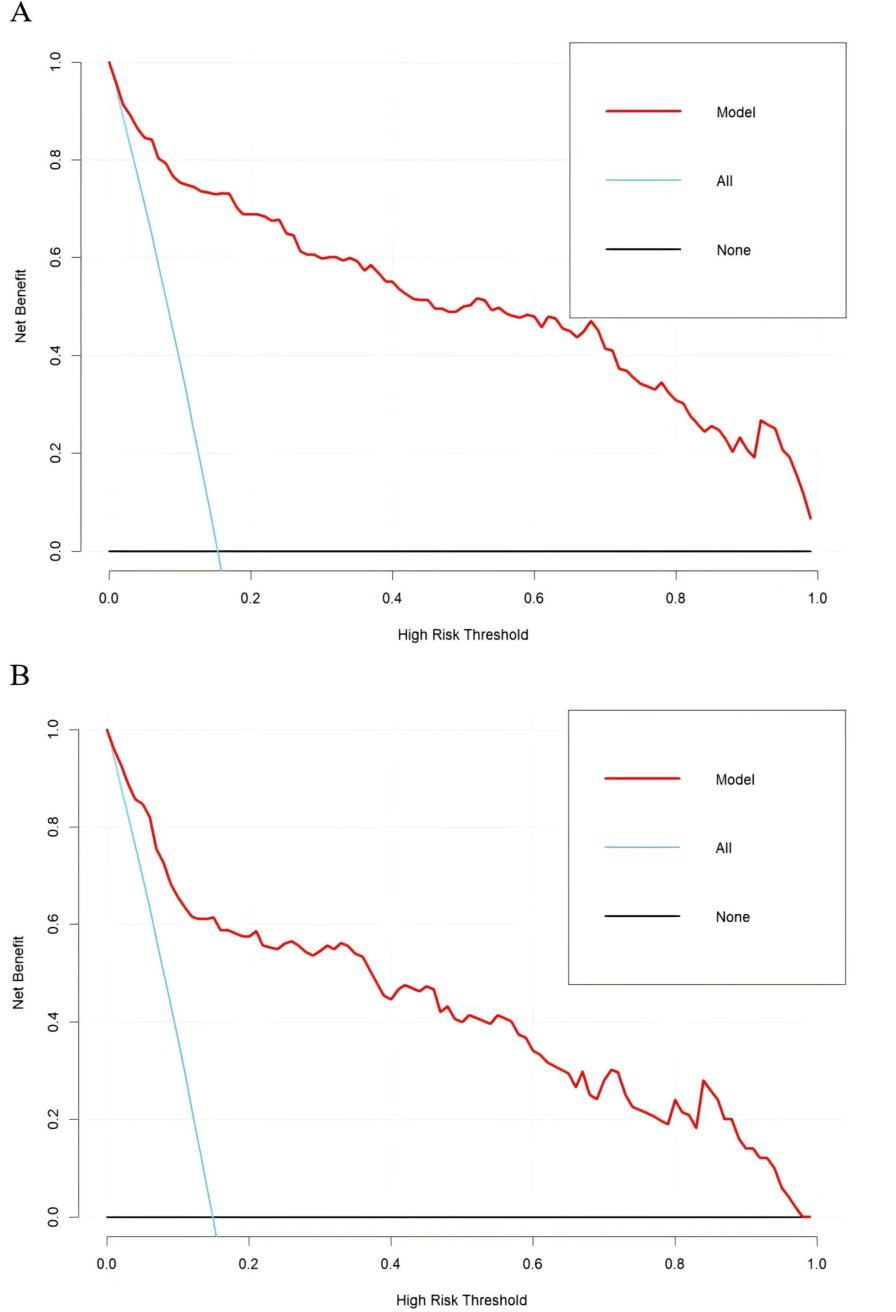
Decision curve analysis for the training group **(A)** and the validation group **(B)**.

The applicability and utility of the model in the training and external validation sets were evaluated by DCA. The DCA curves for both sets showed a significantly higher net benefit than the two extremes, indicating that the nomogram had good clinical benefits (**Figures 6A, B**).

## Discussion

UGIB is a common cause of bleeding after PCI, and in-hospital occurrence of UGIB has a high mortality rate (18). Some patients are more susceptible to have UGIB in one year of dual antiplatelet therapy applied after PCI (19). Consequently, a prediction model that accurately predicts UGIB is beneficial in obtaining a better clinical significance in patients with NSTEMI after PCI. In this study, 1,120 NSTEMI patients with post-PCI were included for analysis and AMR of culprit vessel (≥ 2.5 mmHg*s/cm) were diagnosed with CMD. The results showed that AMR of culprit vessel was an independent predictor of UGIB. In addition, the nomogram constructed by six factors, including HASBLED, TyG index, Alcohol drinking,RBC count, PPI use, and AMR of culprit vessel, can be used to assess the likelihood of UGIB within 1 year in NSTEMI patients undergoing primary PCI.

The mechanisms of CMD may be closely related to inflammation, microvascular spasm, or endothelial dysfunction (20). There is growing evidence that patients with chronic inflammatory diseases (without clinically evident CVD), including psoriasis, psoriatic arthritis, rheumatoid arthritis, and inflammatory bowel disease, have a higher incidence of endothelial and coronary microcirculatory dysfunction (21). In contrast, presence of CMD in NSTEMI patients after PCI may be related to inflammation. In patients with early CAD, coronary segments with macrophage infiltration and vascular proliferation showed a greater response to acetylcholine (ACh) than those without macrophage infiltration and vascular proliferation, suggesting an important role for inflammation and vascular proliferation in the pathogenesis of CAD (22). In addition, the complexity of CMD is further compounded by the possibility of microvascular obstruction (MVO) in patients with acute coronary syndromes (ACS) (23). The causative mechanisms of MVO include distal atherosclerotic thromboembolism, ischemia-reperfusion injury with endothelial cell death along the myocardial cell death, myocardial edema and/or inflammation, which may ultimately result in persistent angina symptoms, despite the fact that the patient has undergone PCI or coronary artery bypass grafting (CABG) (23).

AMR has recently been proposed as a simple way to measure CMD (12). Fan et al. showed a good correlation between AMR and IMR (r=0.83) and found that the accuracy of AMR (≥ 2.5 mmHg*s/cm) for the diagnosis of CMD was high (87.2%) using IMR (≥ 25 U) as the standardized reference (12). In addition, several studies have shown that AMR is not only strongly associated with CMD, but also has a significant association with the prognosis of cardiovascular events. Ma et al. found that AMR was a valid indicator for assessing CMD in patients with obstructive hypertrophic cardiomyopathy, and high microvascular resistance (3 vessel AMR ≥ 7.04) was correlated with poor prognosis (24). And a retrospective study found that AMR could predict the risk of all-cause death or heart failure readmission after PCI in STEMI patients (25). Therefore, AMR, as a surrogate measurement tool for CMD, has an equally important clinical value in the assessment of patient prognosis.

It has been proposed that patients with CMD may not only manifest in the heart, but rather a systemic microvascular disease that may manifest in multi-system disorders including dementia, renal dysfunction, and retinopathy (26). Ohura-Kajitani et al. demonstrated that, in the absence of inhibitors, patients with MVA (Microvascular Angina) and those with both VSA (Vasospastic Angina) and MVA had little or no significant response in resistance arteries to vasodilator drugs compared with patients with VSA alone (27). Overall, the findings support the idea that MVA is not only a microvascular disease limited to the heart, but may also be a systemic microvascular dysfunction, and that MVA can be considered a cardiac manifestation of systemic microvascular disease (27). In patients with CMD, the problem is not limited to the coronary microcirculation and microvascular function can be abnormal throughout the body. Particularly in patients with NSTEMI, one of the possible causes of UGIB after PCI is dysfunction of the GI microcirculation, which may weaken the defenses of the GI mucosa against the acidic environment, thereby increasing the risk of bleeding. Secondly, dysfunction of the heart as a pumping organ may lead to reduced peripheral blood flow, which in turn exacerbates microcirculatory disturbances in the GI tract, leading to decreased mucosal defenses against acidic environments, and ultimately to bleeding. This mechanism provides a potential pathologic explanation for AMR as an independent predictor of UGIB.

Recently, the TyG index has attracted a lot of attention. Several studies have found that the TyG index was an indicator of insulin resistance (IR) and also reflected systemic metabolism (28, 29). A meta-analysis showed an association between a higher TyG index and the risk of developing CAD (30). Shi et al. found a linear positive correlation between TyG index and the occurrence of ischemic stroke (31). Zhao et al. found that the risk of arterial stiffness and renal microcirculatory injury increased with the TyG index (32). In conclusion, a high TyG index was not only a cardiovascular risk factor, but was also associated with cerebrovascular and renal vascular diseases. Although the exact biological mechanisms linking TyG index to disease were unknown, key pathways may be associated with IR. Chronic hyperglycemia and dyslipidemia induced by IR triggers oxidative stress, exacerbates inflammatory responses, promotes foam cell formation, impairs endothelial function, and contributes to smooth muscle cell proliferation (28, 29). In addition, persistent IR increases sympathetic nervous system activity, renal sodium retention, and elevated blood pressure, which increases cardiac load and leads to vascular and renal injury (33). Finally, IR may affect coronary microcirculation, and is strongly associated with myocardial injury and myocardial reperfusion (34). However, few studies have explored the association between TyG index and gastrointestinal circulation. In our study, we found that the TyG index was a predictor of UGIB in NSTEMI patients after PCI, which implied that gastrointestinal circulation was similarly affected in high TyG populations.

Secondly, Feit et al. found that patients with anemia had almost double the risk of all types of bleeding, including a fourfold increase in the risk of GIB, compared to patients without anemia (35). In a two-center study of patients with atrial fibrillation treated with PCI, severe GIB occurred in 12.4% of patients with anemia compared with 3.1% of patients without anemia (36). Similarly, we found that low red blood cell count had a high risk of developing GIB, probably because patients with low red blood cell count were anemic. Patients with anemia often have poor systemic microcirculation, which may affect gastrointestinal microcirculation. In the context of the above discussion, we believe that it is not just damage to the circulation of one organ, but more likely some degree of damage to the systemic microcirculation, of which gastrointestinal hemorrhage is a manifestation.

The HASBLED score was a tool used to assess the risk of bleeding in patients with atrial fibrillation who were receiving anticoagulation therapy (37). In recent years, the application of the HASBLED score has gradually expanded beyond assessing bleeding risk in patients with atrial fibrillation. Konishi et al. and Castini et al. found that the HASBLED score could predict bleeding risk (including GIB) and mortality in patients without atrial fibrillation after PCI (38, 39). HASBLED score incorporated multiple risk factors for GIB (e.g., gender, age, hepatic and renal insufficiency) and we also found that the HASBLED score could be used to predict the occurrence of UGIB after PCI in patients with NSTEMI. A study of patients with CAD treated with dual antiplatelet therapy showed that the higher incidence of UGIB was due to non-administration of PPI and found proton pump inhibitor (PPI) to be more protective than H2 receptor antagonists (H2RA) (40). And in our study, the protective effect of PPI after PCI in patients with NSTEMI was consistent with previous studies. The reason may be that PPI use could selectively inhibit the H+/K+ ATP enzyme in gastric wall cells, significantly reducing gastric acid secretion. This reduction in acid alleviates gastric mucosal damage caused by antiplatelet drugs, as excessive acid secretion could exacerbate mucosal irritation and damage (41).

In summary, we utilized the combination of the classical regression methods and machine learning model to found that HASBLED, TyG index, alcohol drinking, RBC count, PPI use, and AMR of culprit vessel were independent indicators for having UGIB. The model about the relationship between independent factors and UGIB was presented graphically, which had a simple and intuitive effect and the results of this study showed that our prediction model had good performance. While model simplicity reduced the risk of overfitting and improved generalization, the good performance of our current model may not only be a result of the simplicity of the model, but also of the insufficient sample size.

Our study had several limitations. Firstly, this study was a retrospective study and the external data were from the branch hospital. Although AMR is a predictor of UGIB, future prospective studies with large samples are needed to investigate the association between AMR and UGIB and its potential mechanisms. Secondly, not all patients presented to the hospital promptly when signs or tendencies related to bleeding were detected. Thirdly, the collection of UGIB-related risk variables was not comprehensive enough.

## Conclusion

The nomogram clinical prediction model constructed by six factors, including HASBLED, TyG index, alcohol drinking, RBC count, PPI use, and AMR of culprit vessel, can be used to assess the likelihood of UGIB within 1 year in NSTEMI patients undergoing primary PCI, and the nomogram could help clinicians to stratify risk and individualize management for postoperative patients. Secondly this nomogram could also reflect the state of microcirculation throughout the body.

## Data Availability

The raw data supporting the conclusions of this article will be made available by the authors, without undue reservation.
